# Ocorre Lesão Miocárdica após uma Sessão de Exercício Aeróbico Agudo em Pacientes com Angina Refratária?

**DOI:** 10.36660/abc.20210564

**Published:** 2022-11-01

**Authors:** Carla Giuliano de Sá Pinto Montenegro, Luciana Oliveira Cascaes Dourado, Camila Paixão Jordão, Marcelo Luiz Campos Vieira, Camila Regina Alves Assumpção, Luis Henrique Wolff Gowdak, Alexandre da Costa Pereira, Carlos Eduardo Negrão, Luciana Diniz Nagem Janot de Matos

**Affiliations:** 1 Hospital Israelita Albert Einstein São Paulo SP Brasil Hospital Israelita Albert Einstein, São Paulo, SP – Brasil; 2 Hospital das Clínicas da Faculdade de Medicina da Universidade de São Paulo Instituto do Coração São Paulo SP Brasil Instituto do Coração do Hospital das Clínicas da Faculdade de Medicina da Universidade de São Paulo, São Paulo, SP – Brasil; 3 Universidade de São Paulo Escola de Educação Física e Esporte São Paulo SP Brasil Escola de Educação Física e Esporte, Universidade de São Paulo, São Paulo, SP – Brasil

**Keywords:** Angina Pectoris, Exercício Físico, Troponina, Biomarcadores

## Abstract

**Fundamento:**

Não está claro se o exercício é seguro em pacientes com formas mais avançadas de doença arterial coronariana, como aqueles com angina refratária (AR).

**Objetivo:**

Visamos determinar o efeito de uma sessão de exercício aeróbico agudo (SEAA) nos níveis de troponina T cardíaca de alta sensibilidade (TnT-as) em pacientes com AR.

**Métodos:**

Trata-se de um estudo clínico longitudinal, não randomizado e não controlado. Os participantes foram recrutados de abril de 2015 a janeiro de 2019. Em uma escala visual de dor de 0 a 10, a dor classificada até 3 foi considerada como o nível máximo permitido para continuar o exercício. Avaliamos TnT-as na linha de base e 3 horas após a SEAA. O protocolo consistiu em 5 minutos de aquecimento, 30 minutos de exercício aeróbico contínuo na frequência cardíaca correspondente ao limiar anaeróbio ou limiar de angina obtido no teste de esforço cardiopulmonar e 5 minutos de resfriamento. Foram considerados estatisticamente significativos valores de p menores que 0,05.

**Resultados:**

Foram incluídos 32 pacientes com AR (61 ± 9 anos, 59,4% do sexo masculino). A concentração basal de TnT-as foi de 10,9 ng/L (intervalo de confiança de 95%: 9,1 a 13,0 ng/L). A TnT-as coletada 3 horas após a SEAA foi de 11,1 ng/L (intervalo de confiança de 95%: 9,1 a 13,5 ng/L). Nenhuma diferença ocorreu na TnT-as antes e após a SEAA (p = 0,657).

**Conclusões:**

Uma única SEAA realizada no limiar de angina com correspondente escala visual de dor não alterou a TnT-as em pacientes com AR, sugerindo que nenhuma lesão miocárdica significativa foi provocada pelo exercício e que este protocolo de exercício pode ser considerado seguro.

## Introdução

A angina refratária (AR) é uma condição crônica e debilitante caracterizada por angina pectoris com duração > 3 meses, com importante comprometimento da qualidade de vida, como resultado de insuficiência coronariana no quadro de doença arterial que não pode ser controlada por terapia medicamentosa em pacientes não elegíveis para revascularização coronária (cirurgia ou angioplastia). Apesar da complexidade da doença, esses pacientes apresentam baixa incidência de eventos combinados, incluindo uma incidência de morte de aproximadamente 2% a 3% ao ano e uma incidência de infarto do miocárdio de 3,5% ao ano.^1^

Evidências crescentes mostram que o exercício é uma estratégia importante no tratamento de pacientes com doença arterial coronariana (DAC).^2^ Demonstrou-se que o exercício aumenta o fluxo sanguíneo coronariano e periférico.^2^ Da mesma forma, o treinamento físico melhora o controle neurovascular em pacientes com DAC,^3^ o que parece estar associado à melhora do controle barorreflexo arterial. Além disso, o treinamento de exercício melhora a tolerância ao exercício e a qualidade de vida^4^ e reduz a mortalidade nesse grupo de pacientes.^5^ Ainda não está claro se o exercício é seguro em pacientes com formas mais avançadas de DAC, como aqueles com AR, porque, pelo menos teoricamente, o exercício poderia provocar isquemia grave e/ou prolongada, levando a danos no miocárdio.

A troponina T cardíaca de alta sensibilidade (TnT-as) é um marcador específico de lesão miocárdica.^6^ Além disso, essa proteína está relacionada ao prognóstico de pacientes com AR. Estudos anteriores mostraram que a TnT-as é um preditor significativo de mortalidade e infarto do miocárdio não fatal.^7^ Alguns pesquisadores têm sugerido que a elevação da TnT-as após o exercício físico é um sinal de isquemia induzida pelo exercício,^8–10^ que depende da intensidade e da duração do exercício.^10^

No presente estudo, relatamos os níveis de TnT-as após uma sessão de exercício aeróbico agudo (SEAA) em pacientes com AR. Nós hipotetizamos que o exercício de intensidade moderada feito no limiar anaeróbico ou limiar de angina confirmado por uma escala visual subjetiva de dor não alteraria os níveis de TnT-as neste grupo de pacientes.

## Métodos

Trata-se de um estudo clínico longitudinal, não randomizado e não controlado, realizado para avaliar as respostas à SEAA programada em pacientes com AR acompanhados em um hospital universitário terciário. A presente análise fez parte do estudo intitulado “Reabilitação Cardíaca em Pacientes com Angina Refratária”. O tamanho da amostra foi definido por conveniência. O estudo foi aprovado pelo comitê de ética e pesquisa do Hospital das Clínicas da Faculdade de Medicina da Universidade de São Paulo (CAAE: 24308213.7.0000.0068), submetido e aprovado no Clinical Trials (NCT03218891). As investigações seguiram a Declaração de Helsinque. Todos os pacientes forneceram consentimento informado por escrito.

### Seleção de pacientes

De abril de 2015 a janeiro de 2019, foram incluídos pacientes entre 45 e 75 anos de idade, de ambos os sexos, com angina sintomática (classe funcional de angina da Canadian Cardiovascular Society [CCS] de II a IV) com pelo menos 3 meses de duração sob terapia medicamentosa otimizada, que não eram elegíveis para procedimentos cirúrgicos ou de revascularização miocárdica percutânea e nos quais foi possível documentar a isquemia miocárdica por ecocardiografia de estresse físico.

### Critérios de exclusão

Os critérios de exclusão foram: 1) marca-passos permanentes ou desfibriladores cardíacos implantáveis; 2) pacientes com ritmo não sinusal; 3) histórico recente (< 3 meses) de síndrome coronariana aguda ou revascularização miocárdica (percutânea ou cirúrgica); 4) comprometimento funcional causado por qualquer condição clínica que impeça o exercício; e 5) restrição de atividade (classe D) de acordo com os critérios da American Heart Association para estratificação de risco de eventos durante o exercício.^7^

### Teste de exercício cardiopulmonar

O teste de exercício cardiopulmonar (TECP) foi realizado em esteira motorizada (modelo T2100, GE Healthcare, EUA) utilizando um protocolo de exercício incremental (Balke 2,5 mph). A frequência cardíaca (FC) foi registrada continuamente por meio de eletrocardiograma de 12 derivações (Ergo PC, Micromed, Brasil). As saídas de oxigênio e dióxido de carbono foram medidas por análise respiração-a-respiração (SensorMedics, VmaxAnalyzer Assembly, Encore 29S, EUA). Foi realizado o TECP seguindo as diretrizes, assim como os critérios para definição do esforço máximo e a determinação do limiar anaeróbio. O limiar de angina foi determinado na FC exata em que o paciente se queixou de sintomas de angina.

### Análise da troponina T cardíaca de alta sensibilidade

Foi utilizado um ensaio de alta sensibilidade comercialmente disponível para cTnT (Troponin T, kit Elisa, Roche Diagnostics, Alemanha), com um limite de detecção de 3 ng/L e um ponto de corte do percentil 99 de uma população de referência aparentemente saudável de < 14 ng/mL. Considerando que o pico de liberação de TnT-as induzida pelo exercício ocorre nas primeiras 1 a 4 horas,^11^ medimos o nível de TnT-as 3 horas após a SEAA.

### Escala de dor

Uma escala visual numérica de dor foi adotada e utilizada tanto para determinar a interrupção do exercício quanto para reduzir a sua intensidade. A escala foi graduada de 0 (sem dor) a 10 (dor intensa). A dor classificada até 3 (leve a moderada) foi considerada como o nível máximo permitido para continuar o exercício aeróbico. Quando a dor atingiu intensidade superior a 3 (moderada), o exercício foi interrompido ou sua intensidade foi reduzida.

### Protocolo da sessão de exercício aeróbico agudo

O protocolo consistiu em um total de 40 minutos de exercício: 5 minutos de aquecimento, 30 minutos de exercício aeróbico contínuo em esteira motorizada na FC correspondente ao limiar anaeróbio ou limiar de angina obtido no TECP e 5 minutos de desaquecimento. Foi recomendado exercício contínuo; entretanto, interrupções breves ou redução de intensidade foram permitidas se o paciente apresentasse angina moderada ou percepção moderada de esforço. O exercício foi reiniciado quando os sintomas não foram mais observados. Os pacientes foram monitorados continuamente por telemetria. A administração de 5 mg de dinitrato de isossorbida sublingual foi administrada conforme necessário.

### Análise estatística

As variáveis contínuas são expressas como média ± desvio padrão ou mediana e intervalo interquartil (IIQ), conforme apropriado, e as variáveis categóricas são expressas como frequências absolutas e relativas. As distribuições das variáveis numéricas foram estudadas por meio de histogramas e *boxplots*, bem como por testes de normalidade de Shapiro-Wilk. Modelos de equações de estimativa generalizada foram ajustados para investigar variações entre as avaliações antes e após uma sessão de exercício físico para comparar grupos com e sem AR, uso de nitrato durante a sessão de exercício físico e classe funcional da CCS, em relação à dosagem de troponina ultrassensível. Foram realizados ajustes com distribuição gama e função de ligação logarítmica, considerando a correlação entre as medidas de um mesmo paciente nos dois momentos de avaliação. Para comparar a presença de angina em relação às variações observadas nas dosagens de troponina, foi utilizado o teste não paramétrico de Mann-Whitney. As correlações entre as medidas numéricas foram investigadas por meio do coeficiente de correlação de Spearman. Um modelo linear generalizado com distribuição gama e função de ligação logarítmica foi ajustado para comparar os grupos com e sem angina durante a sessão de exercício físico em relação à FC naquela sessão. As análises foram realizadas usando o pacote estatístico SPSS para Windows, versão 20.0 (IBM Corp), considerando um nível de significância de 5%.

## Resultados

### Medidas basais

Foram recrutados 92 pacientes e 60 deles foram excluídos por não atenderem aos critérios de inclusão. O estudo incluiu 32 pacientes com AR (61 ± 9 anos, 59,4% do sexo masculino), 40,6% com classe funcional de angina da CCS II, 21,9% com classe III e 37,5% com classe IV. As características clínicas dos pacientes e as medicações antianginosas utilizadas são apresentadas na Tabela 1. A maioria dos pacientes (90,6%) fazia uso de uma combinação de pelo menos 3 antianginosos, 93,8% aspirina, 100% estatina e 34,4% insulina. A análise do TECP demonstrou um tempo total de exercício médio de 344,5 ± 139,5 segundos. A FC do limiar de angina e do limiar anaeróbio foi de 88,5 ± 15,9 batimentos por minuto (bpm) e 84,6 ± 11,9 bpm, respectivamente. O consumo de oxigênio de pico foi de 16,1 ± 3,4 mL/kg/min.

**Tabela 1:** Características demográficas e clínicas basais dos pacientes

Dados clínicos		(N=32)
Idade, anos (média ± DP)		61±9
Sexo masculino (%)		59,4
IMC, kg/m^2^ (média ± DP)		29,1±4,0
CA, cm (média ± DP)		100,6±10,0
FC em repouso, bpm (média ± DP)		61±7
PAS, mmHg (média ± DP)		124±16
PAD, mmHg (média ± DP)		77±11
**Histórico médico prévio (%)**		
	Hipertensão	75,0
	Diabetes mellitus	71,9
	Hiperlipidemia	93,8
	Tabagismo prévio	71,9
	Obesidade	34,4
	Histórico familiar de DAC	56,3
	Infarto agudo do miocárdio prévio	75,0
Tempo diagnóstico das DAC, anos (média ± DP)		12±9
FEVE ecocardiográfica, % (média ± DP)		54±9
**Padrão obstrutivo de DAC (%)**		
	Doença de 1 vaso	6,3
	Doença de 2 vasos	12,5
	Doença de 3 vasos	81,3
**Achados laboratoriais (média ± DP)**		
	LDL-colesterol, mg/dL	78,0±26,5
	HDL-colesterol, mg/dL	44,3±12,9
	Triglicerídeos, mg/dL	129,6±54,3
	HBA1C, %	7,2±1,7
**Uso de medicamentos antianginosos (%)**		
	Betabloqueadores	100
	Bloqueadores dos canais de cálcio	84,4
	Nitratos de ação prolongada	93,8
	Trimetazidina	96,9
	Ivabradina	15,6

CA: circunferência abdominal; DAC: doença arterial coronariana; DP: desvio padrão; FC: frequência cardíaca; FEVE: fração de ejeção do ventrículo esquerdo; IMC: índice de massa corporal; PAD: pressão arterial diastólica; PAS: pressão arterial sistólica.

A análise da isquemia documentada pelo ecocardiograma de estresse demonstrou que 62,5% dos pacientes apresentavam isquemia na artéria descendente anterior esquerda, 41% na artéria circunflexa e 37% nos territórios da artéria coronária direita. A maioria (66%) dos pacientes apresentou isquemia em apenas 1 território coronariano, 28% em 2 territórios e 6% em 3 territórios.

### Respostas ao exercício

A concentração basal de TnT-as foi de 10,9 ng/L (intervalo de confiança [IC] de 95%: 9,1 a 13,0 ng/L) e 3 horas após a sessão de exercício foi de 11,1 ng/L (IC 95%: 9,1 a 13,5 ng/L). Não foram encontradas diferenças significativas nas dosagens de TnT-as antes e após a sessão (p = 0,657, Tabela 2). Os níveis de TnT-as diminuíram em 21,9% dos pacientes, não se alteraram em 31,2% e aumentaram em 46,9%.

**Tabela 2:** Valores médios estimados e intervalos de confiança de 95% para dosagem de troponina ultrassensível antes e após uma sessão de exercício (n = 32)

Avaliação	Dosagem de troponina ultrassensível (ng/L)
Antes da SE	10,9 (9,1; 13,0)
Após a SE	11,1 (9,1; 13,5)
Variação (após a SE – antes da SE)	0,2 (–0,8; 1,2)
Valor p	0,657

Valores expressos como médias estimadas (intervalo de confiança de 95%); SE: sessão de exercício. Teste estatístico: modelos de equações de estimativa generalizada

**Figura 1:**
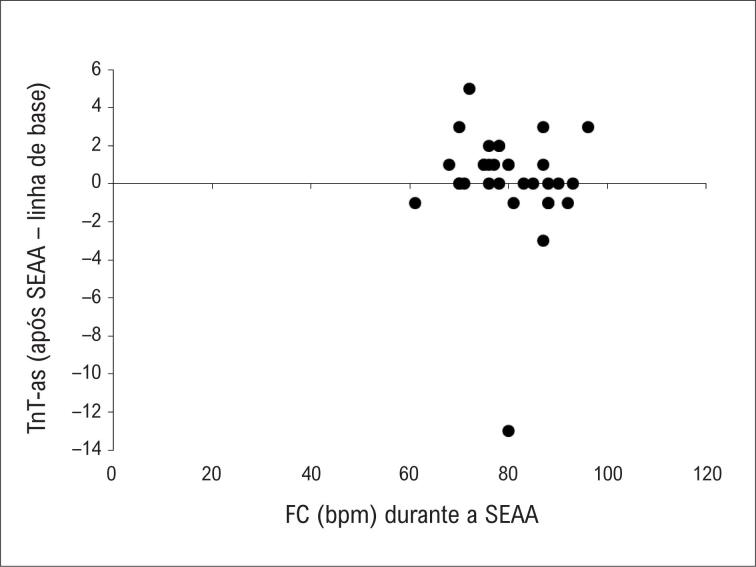
Relação entre FC da SEAA e delta da TnT-as. bpm: batimentos por minuto; FC: frequência cardíaca; SEAA: sessão de exercício aeróbico agudo; TnT-as: troponina T cardíaca de alta sensibilidade.

A média da FC mantida durante a SAAE foi de 82,8 ± 7,8 bpm, com 37,5% dos pacientes atingindo a FC do limiar anaeróbio. Durante a sessão, 53,1% dos pacientes apresentaram angina na média de 81,2 ± 8,5 bpm e, destes, 52,9% não atingiram o limiar de angina do TECP. Por outro lado, dos pacientes que não apresentaram angina durante a SEAA, 40% atingiram o limiar de angina do TECP. Os pacientes que apresentaram angina durante a SEAA tiveram FC da SEAA significativamente maior (diferença média: 6,3 bpm) do que aqueles que não apresentaram (p = 0,018; IC 95%, 1,1 a 11,5 bpm). No entanto, não foi observada correlação entre a FC da SEAA e os valores de delta do nível de TnT-as (R = −0,25; p = 0,176) (Figura 1). Os valores de delta das concentrações de TnT-as não diferiram entre os pacientes que apresentaram angina durante a sessão e aqueles que não apresentaram (medianas de 0,0 [IIQ: 0,0 a 2,0] e 1,0 [IIQ: −1,0 a 1,0], respectivamente [p = 0,941]).

Apenas 12,5% de todos os pacientes necessitaram de nitrato sublingual de curta duração para alívio da angina durante a SEAA, e destes, apenas 1 (25%) atingiu o limiar de angina do TECP durante a sessão. Não ocorreram diferenças significativas entre os níveis de TnT-as e o uso de nitrato durante a SEAA (p = 0,077) ou entre os níveis de TnT-as e a classe funcional de angina da CCS dos pacientes (p = 0,395). Nenhum evento adverso ocorreu durante a SEAA ao longo do estudo.

## Discussão

Até onde sabemos, este é o primeiro estudo a especificamente investigar lesão miocárdica por TnT-as após uma única sessão de exercício em pacientes com AR. Nossos resultados demonstram que uma SEAA realizada até o limiar de angina correspondente ao nível 3 na escala visual de dor de 0 a 10 não alterou a concentração de TnT-as nem causou complicações clínicas em pacientes com AR. Portanto, o exercício físico pode ser indicado de forma mais abrangente como parte do tratamento não farmacológico desses pacientes.

Esses resultados são um passo importante na prática clínica, pois uma das características clínicas mais importantes dos pacientes com AR é a baixa tolerância ao exercício, limitando suas atividades diárias e piorando sua qualidade de vida.^12^ Nesse contexto, o exercício físico poderia ser incluído como parte do tratamento clínico seguro da AR. Habitualmente, esses pacientes apresentam restrições a atividades físicas devido ao medo de que o exercício possa exacerbar os sintomas ou desencadear eventos cardiovasculares recorrentes. A reabilitação cardíaca, tratamento estabelecido para pacientes com DAC (classe I, nível de evidência A),^13,14^ não é claramente recomendada para pacientes com AR, devido à falta de evidências quanto à segurança e aos efeitos benéficos.^15,16^

A TnT-as é um biomarcador reconhecido para diagnóstico de infarto do miocárdio e lesão miocárdica.^17,18^ As concentrações de troponina, mesmo abaixo do percentil 99, predizem resultados adversos em pacientes e na população geral.^19^ Em pacientes com DAC, incluindo aqueles com AR,^1^ a TnT-as é um importante marcador prognóstico de eventos cardiovasculares.^1,20,21^ A baixa TnT-as circulante foi identificada como o mais forte preditor de morte e lesão miocárdica não fatal. Uma possível explicação se deve à ocorrência de rupturas silenciosas de placas ateroscleróticas vulneráveis, que levam à microembolização e subsequentemente ao microinfarto em áreas do miocárdio não supridas por circulação colateral suficiente, elevando os níveis plasmáticos de TnT-as antes da ocorrência de lesão miocárdica e morte cardiovascular.^7^

As troponinas cardíacas também podem ser liberadas com o exercício; porém, os mecanismos subjacentes e o valor clínico real desse aumento não são completamente compreendidos.^8,10,22^ Várias teorias têm sido propostas para explicar o mecanismo subjacente à liberação de troponina, seguida em muitos casos por anormalidades da fração de ejeção documentadas por ecocardiografia.^23^ A lesão miocárdica é o mais preocupante dos mecanismos propostos, pois pode resultar em desfecho clínico negativo. Outro mecanismo bem aceito é a liberação de uma troponina não ligada que existe no citosol do cardiomiócito através do aumento da permeabilidade da membrana do cardiomiócito (desencadeada pelo estresse de cisalhamento do exercício). Isso poderia explicar a curta duração (< 1 semana) do aumento da TnT-as após o exercício.

A magnitude da resposta da TnT-as depende da duração e, principalmente, da intensidade da atividade física, e o significado prognóstico de longo prazo da liberação repetitiva de troponina induzida pelo exercício não é completamente conhecido.^10,22^ Muitos autores consideram benignos esses aumentos nas concentrações de troponina induzidos pelo exercício, pois ocorrem com frequência, estão presentes mesmo em indivíduos (aparentemente) saudáveis e não são acompanhados de sintomas clínicos.^24–26^ Por outro lado, um estudo recente observou que TnT-as basal e pós-exercício foram preditores independentes de mortalidade e eventos cardiovasculares adversos maiores em uma coorte de idosos caminhantes de longa distância, chamando a atenção para o fato de que altos níveis de troponina podem não ser apenas um resposta fisiológica benigna, mas um marcador prognóstico precoce de eventos cardiovasculares.^19^

Considerando a falta de consenso sobre a relevância clínica da TnT-as pós-exercício no prognóstico, a interpretação desses estudos deve levar os seguintes fatores em consideração: 1) a população avaliada, 2) a intensidade e o volume de exercício aplicado, 3) os ensaios de medida de TnT-as, 4) a experiência de treinamento, 5) o tempo de coleta de sangue e 6) a presença de sintomas durante o exercício.^10,22^

Diante desses pontos controversos e dos nossos achados, parece que a atividade física, especialmente em pacientes com DAC, pode ser praticada com segurança quando não é seguida por aumento da TnT-as pós-exercício, orientando a prescrição de uma “dose-alvo otimizada”. Dessa maneira, minimiza os possíveis efeitos nocivos do exercício, alcançando benefícios máximos para a saúde e, adicionalmente, mudando os paradigmas de limitar a prescrição de exercícios abaixo do limiar de isquemia ou de angina.

O treinamento físico baseado no limiar de dor associado à escala visual de dor é amplamente utilizado e aceito em pacientes com doença arterial periférica.^27^ Nesses pacientes, esse tipo de treinamento é considerado uma forma de tratamento.^27^ Em pacientes com DAC, o exercício é realmente recomendado na intensidade abaixo da isquemia induzível. A intensidade do exercício aeróbio deve ser prescrita 10 batimentos abaixo do limiar de isquemia, geralmente controlada por alterações eletrocardiográficas.^7^ Em pacientes com AR, que são extremamente limitados na realização de tarefas diárias devido à angina e cujos sintomas são desencadeados por estressores físicos e emocionais, é um desafio definir a FC baseada no exercício.

Em nossos pacientes com AR, 25% apresentaram TnT-as acima do percentil 99 da normalidade (> 14 ng/L), ou seja, com maior risco de eventos cardiovasculares. Apesar disso e da presença de angina moderada (até 3 de 10 na escala visual de dor), em mais da metade dos pacientes, não observamos aumento significativo de TnT-as após SEAA ou eventos clínicos durante a sessão. A angina, como outras dores crônicas, é um sintoma subjetivo e muito individual e, obviamente, deve ser levado em consideração para a segurança desses pacientes. É essencial destacar que nossos pacientes não apresentaram alterações eletrocardiográficas durante a sessão de exercício, apesar de apresentarem angina. Portanto, a prescrição de intensidade de exercício baseada em modificações eletrocardiográficas não parece ser uma estratégia adequada em pacientes com AR.

É necessário ressaltarmos que os pacientes que apresentaram angina durante a sessão de exercício apresentaram FC mais elevada do que os pacientes sem angina. Porém, os pacientes sem angina treinaram até o limiar anaeróbio ou esforço moderado, que é a intensidade de exercício recomendada para pacientes com DAC. Esses resultados reforçam que nossa proposta de prescrição de exercícios pode ser considerada segura para aplicação em pacientes com AR.

### Limitações

A análise da TnT-as foi feita com base em amostras em 2 pontos: antes e 3 horas após a SEAA. A última foi definida com base na evidência de que qualquer alteração no nível de TnT-as já é detectável naquele momento.^17,28^ Portanto, não acreditamos que tenha interferido em nossos resultados.

## Conclusão

Uma única SEAA realizada no limiar de angina com correspondente escala visual de dor (até 3 de 10) não alterou a TnT-as em pacientes com AR, sugerindo que nenhuma lesão miocárdica significativa foi provocada pelo exercício. Portanto, concluímos que este protocolo de exercício pode ser considerado seguro para pacientes com AR.

### O que se sabe sobre o assunto e o que este estudo acrescenta?

O treinamento físico melhora a tolerância ao exercício e a qualidade de vida e reduz a mortalidade nesse grupo de pacientes; entretanto, a segurança da atividade física ainda é questionada para pacientes com angina refratária e muitas vezes é um impedimento para a realização de reabilitação cardíaca, impactando assim na qualidade de vida desta população. Este manuscrito cria um paradigma para estudos futuros de reabilitação em pacientes com angina refratária.
