# Supplementary material for: Ocorre Lesão Miocárdica após uma Sessão de Exercício Aeróbico Agudo em Pacientes com Angina Refratária?
Source: Arq Bras Cardiol. 2022 Nov 1;119(5):747–53. [Article in Portuguese] doi: 10.36660/abc.20210564 (PMC9750210; doi:10.36660/abc.20210564)
Supplement: Translation

# Translation: Does Myocardial Injury Occur After an Acute Aerobic Exercise Session in Patients with Refractory Angina?

**Authors:** Carla Giuliano de Sá Pinto Montenegro, Luciana Oliveira Cascaes Dourado, Camila Paixão Jordão, Marcelo Luiz Campos Vieira, Camila Regina Alves Assumpção, Luis Henrique Wolff Gowdak, Alexandre da Costa Pereira, Carlos Eduardo Negrão, Luciana Diniz Nagem Janot de Matos

**Affiliations:** 1 São Paulo SP Brazil Hospital Israelita Albert Einstein, São Paulo, SP – Brazil; 2 São Paulo SP Brazil Instituto do Coração do Hospital das Clínicas da Faculdade de Medicina da Universidade de São Paulo, São Paulo, SP – Brazil; 3 São Paulo SP Brazil Escola de Educação Física e Esporte, Universidade de São Paulo, São Paulo, SP – Brazil

**Keywords:** Angina Pectoris, Exercise, Troponin, Biomarkers

## Abstract

**Background:**

It is unclear whether exercise is safe in patients with more advanced forms of coronary artery disease, such as those with refractory angina (RA).

**Objective:**

We aimed to determine the effect of an acute aerobic exercise session (AAES) on high-sensitivity cardiac troponin T (hs-cTnT) levels in patients with RA.

**Methods:**

This was a longitudinal, non-randomized, and non-controlled clinical study. Participants were recruited from April 2015 to January 2019. On a visual pain scale from 0 to 10, pain rated up to 3 was considered as the top level allowed to continue exercising. We assessed hs-cTnT at baseline and 3 hours after the AAES. The protocol consisted of 5 minutes of warm-up, 30 minutes of continuous aerobic exercise at heart rate corresponding to the anaerobic threshold or angina threshold obtained in the cardiopulmonary exercise testing, and 5 minutes of cooling down. P values less than 0.05 were considered statistically significant.

**Results:**

Thirty-two patients with RA were included (61 ± 9 years, 59.4% male). The baseline hs-cTnT concentration was 10.9 ng/L (95% confidence interval: 9.1 to 13.0 ng/L). The hs-cTnT collected 3 hours after the AAES was 11.1 ng/L (95% confidence interval: 9.1 to 13.5 ng/L). No difference occurred in hs-cTnT before and after AAES (p = 0.657).

**Conclusions:**

A single AAES performed at the angina threshold with corresponding visual pain scale did not alter hs-cTnT in patients with RA, suggesting that no significant myocardial injury was elicited by exercising and that this exercise protocol can be considered safe.

## Introduction

Refractory angina (RA) is a chronic and debilitating condition characterized by angina pectoris lasting > 3 months, with an important impairment of quality of life, as a result of coronary insufficiency in the setting of artery disease that cannot be controlled by medical therapy in patients who are not eligible for coronary revascularization (surgery or angioplasty). Despite the complexity of the disease, these patients present low incidence of combined events, including an approximately 2% to 3% per year incidence of death and a 3.5% per year incidence of myocardial infarction.^1^

Growing evidence shows that exercise is an important strategy in the treatment of patients with coronary artery disease (CAD).^2^ Exercise has been shown to increase coronary and peripheral blood flow.^2^ Likewise, exercise training improves neurovascular control in patients with CAD,^3^ which seems to be associated with improvement in arterial baroreflex control. Moreover, exercise training improves exercise tolerance and quality of life^4^ and reduces mortality in this group of patients.^5^ It is still unclear whether exercise is safe in patients with more advanced forms of CAD, such as those with RA, because, at least theoretically, exercise could provoke severe and/or prolonged ischemia, leading to myocardial damage.

High-sensitivity cardiac troponin T (hs-cTnT) is a specific myocardial injury (MI) marker.^6^ Also, this protein is related to the prognosis of patients with RA. Previous studies have shown that hs-cTnT is a significant predictor of mortality and nonfatal myocardial infarction.^7^ Some investigators have suggested that elevation of hs-cTnT after physical exercise is a sign of exercise-induced ischemia,^8–10^ which depends upon exercise intensity and duration.^10^

In this study, we report the levels of hs-cTnT after an acute aerobic exercise session (AAES) in patients with RA. We hypothesized that moderate-intensity exercise done at the anaerobic threshold or angina threshold confirmed by a subjective visual pain scale would not alter the levels of hs-cTnT in this group of patients.

## Methods

This was a longitudinal, non-randomized, and non-controlled clinical study performed to evaluate the responses to programmed AAES in patients with RA followed in a tertiary university hospital. This analysis was part of the study entitled “Cardiac Rehabilitation in Patients with Refractory Angina”. The sample size was defined by convenience. The study was approved by the ethics and research committee of the Hospital das Clínicas da Faculdade de Medicina da Universidade de São Paulo (CAAE: 24308213.7.0000.0068), submitted to and approved in Clinical Trials (NCT03218891). Investigations followed the Declaration of Helsinki. All patients provided written informed consent.

### Patient selection

From April 2015 to January 2019, we enrolled patients of both sexes, aged 45 to 75 years, with symptomatic angina (Canadian Cardiovascular Society [CCS] angina functional class II to IV) with at least 3 months of duration on optimal medical therapy, in whom myocardial ischemia could be documented by physical stress echocardiography, and who were not eligible for surgical or percutaneous myocardial revascularization procedures.

### Exclusion criteria

Exclusion criteria were as follows: 1) permanent pacemakers or implantable cardiac defibrillators; 2) patients with nonsinus rhythm; 3) history of recent (< 3 months) acute coronary syndrome or myocardial revascularization (percutaneous or surgical); 4) functional impairment caused by any clinical condition preventing exercise; and 5) activity restriction (class D) according to the American Heart Association criteria for risk stratification of events during exercise.^7^

### Cardiopulmonary exercise testing

The cardiopulmonary exercise test (CPET) was performed on a motorized treadmill (T2100 model, GE Healthcare, USA) using a graded exercise protocol (Balke 2.5 mph). Heart rate (HR) was continuously recorded using a 12-lead electrocardiogram (Ergo PC, Micromed, Brazil). The oxygen and carbon dioxide outputs were measured by breath-by-breath analysis (SensorMedics, VmaxAnalyzer Assembly, Encore 29S, USA). CPET was performed following the guidelines, as well as the criteria for defining maximal effort and determination of the anaerobic threshold. The angina threshold was determined at the exact HR at which the patient complained of angina symptoms.

### High-sensitivity cardiac troponin T analysis

A commercially available high-sensitivity assay for cTnT (Troponin T, Elisa kit, Roche Diagnostics, Germany), with a detection limit of 3 ng/L and a 99th percentile cut-off from an apparently healthy reference population of < 14 ng/mL was utilized. Because exercise-induced hs-cTnT peak release occurs within the first 1 to 4 hours,^11^ we measured the hs-cTnT level at 3 hours post-AAES.

### Pain scale

A visual numeric pain scale was adopted and used either to determine exercise interruption or to reduce its intensity. The scale was graded from 0 (no pain) to 10 (severe pain). Pain rated up to 3 (mild to moderate) was considered as the top level allowed to continue aerobic exercise. When the pain reached an intensity higher than 3 (moderate), exercise was interrupted, or its intensity was reduced.

### Acute aerobic exercise session protocol

The protocol consisted of a total of 40 minutes exercising: 5 minutes of warm-up, 30 minutes of continuous aerobic exercise on a motorized treadmill at HR corresponding to the anaerobic threshold or angina threshold obtained in the CPET, and 5 minutes of cool-down. Continuous exercise was recommended; however, brief interruptions or reduction in intensity were allowed if the patient experienced moderate angina or moderate effort perception. The exercise was restarted when the symptoms were no longer observed. Patients were continuously monitored by telemetry. Administration of 5 mg of sublingual isosorbide dinitrate was administered as needed.

### Statistical analysis

Continuous variables are expressed as mean ± standard deviation or median and interquartile range (IQR) as appropriate, and categorical variables are expressed as absolute and relative frequencies. The distributions of numerical variables were studied using histograms and boxplots, as well as by Shapiro-Wilk normality tests. Generalized estimating equation models were adjusted to investigate variations between the assessments before and after a physical exercise session to compare groups with and without RA, nitrate use during the physical exercise session, and CCS functional class, in relation to the dosage of ultrasensitive troponin. Adjustments were performed with gamma distribution and logarithmic linkage function, considering the correlation between the measurements of the same patient in both evaluation moments. To compare the presence of angina in relation to the variations observed in troponin dosages, the non-parametric Mann-Whitney test was used. Correlations between numerical measures were investigated using Spearman's correlation coefficient. A generalized linear model with gamma distribution and logarithmic link function was fitted to compare groups with and without angina during the physical exercise session in relation to HR during that session. The analyses were performed using the SPSS statistical package for Windows, version 20.0 (IBM Corp), considering a significance level of 5%.

## Results

### Baseline measures

A total of 92 patients were recruited, and 60 of them were excluded for not meeting the inclusion criteria. The study included 32 patients with RA (61 ± 9 years, 59.4% male), 40.6% with CCS II angina functional class, 21.9% with CCS III, and 37.5% with CCS IV. The clinical characteristics of the patients and antianginal medications used by patients are shown in Table 1. Most patients (90.6%) were on a combination of at least 3 antianginal drugs, 93.8% aspirin, 100% statin, and 34.4% insulin. The CPET analysis demonstrated a mean total exercise time of 344.5 ± 139.5 seconds. HR of angina threshold and anaerobic threshold were 88.5 ± 15.9 beats per minute (bpm) and 84.6 ± 11.9 bpm, respectively. Peak oxygen consumption was 16.1 ± 3.4 mL/kg/min.

**Table 1:** Baseline demographic and clinical characteristics of patients

Clinical data		(N=32)
Age, years (mean±SD)		61±9
Male (%)		59.4
BMI, kg/m^2^ (mean±SD)		29.1±4.0
AC, cm (mean±SD)		100.6±10.0
Resting HR, bpm (mean±SD)		61±7
SBP, mmHg (mean±SD)		124±16
DBP, mmHg (mean±SD)		77±11
**Past medical history (%)**		
	Hypertension	75.0
	Diabetes mellitus	71.9
	Hyperlipidemia	93.8
	Previous smoking	71.9
	Obesity	34.4
	Family history of CAD	56.3
	Previous acute myocardial infarction	75.0
CAD diagnostic time, years (mean±SD)		12±9
Echocardiographic LVEF, % (mean±SD)		54±9
**CAD obstructive pattern (%)**		
	One-vessel disease	6.3
	Two-vessel disease	12.5
	Three-vessel disease	81.3
**Laboratory findings (mean±SD)**		
	LDL-cholesterol, mg/dL	78.0±26.5
	HDL-cholesterol, mg/dL	44.3±12.9
	Triglycerides, mg/dL	129.6±54.3
	HBA1C, %	7.2±1.7
**Antianginal drugs in use (%)**		
	β-blockers	100
	Calcium channel blockers	84.4
	Long-acting nitrates	93.8
	Trimetazidine	96.9
	Ivabradine	15.6

AC: abdominal circumference; BMI: body mass index; CAD: coronary artery disease; DBP: diastolic blood pressure; HR: heart rate; LVEF: left ventricular ejection fraction; SBP: systolic blood pressure; SD: standard deviation.

The analysis of ischemia documented by the stress echocardiogram demonstrated that 62.5% of the patients had ischemia in the left anterior descending artery, 41% in the circumflex artery, and 37% in the right coronary artery territories. Most (66%) patients had ischemia in only 1 coronary territory, 28% in 2 territories, and 6% in 3 territories.

### Responses to exercise

The baseline hs-cTnT concentration was 10.9 ng/L (95% confidence interval [CI]: 9.1 to 13.0 ng/L), and 3 hours after the exercise session it was 11.1 ng/L (95% CI: 9.1 to 13.5 ng/L). No significant differences were found in hs-cTnT dosages before and after the session (p =0.657, Table 2). The hs-cTnT levels decreased in 21.9% of patients, did not change in 31.2%, and increased in 46.9%.

**Table 2:** Estimated mean values and 95% confidence intervals for ultrasensitive troponin dosage before and after an exercise session (n=32)

Assessment	Ultrasensitive troponin dosage (ng/L)
Before EX	10.9 (9.1; 13.0)
After EX	11.1 (9.1; 13.5)
Variation (after EX –before EX)	0.2 (–0.8; 1.2)
p value	0.657

Values expressed as estimated averages (95% confidence interval); EX: exercise session. Statistical test: generalized estimating equation models.

The mean HR maintained during AAES was 82.8 ± 7.8 bpm, with 37.5% of patients achieving the HR of anaerobic threshold. During the session, 53.1% of patients experienced angina at the mean of 81.2 ± 8.5 bpm, and, of these, 52.9% did not achieve CPET angina threshold. On the other hand, of the patients who did not have angina during AAES, 40% achieved a CPET angina threshold. Patients who experienced angina during AAES had a significantly higher AAES HR (mean difference: 6.3 bpm) than those who did not (p = 0.018; 95% CI, 1.1 to 11.5 bpm). However, no correlation was noted between AAES HR and delta hs-cTnT levels (R = −0.25; p = 0.176) (Figure 1). The delta hs-cTnT concentrations did not differ between patients who experienced angina during the session and those who did not (medians of 0.0 [IQR: 0.0 to 2.0] and 1.0 [IQR: −1.0 to 1.0], respectively [p = 0.941]).

**Figure 1:** Relationship between AAES HR and delta hs-cTnT. AAES: acute aerobic exercise session; bpm: beats per minute; HR: heart rate; hs-cTnT: high-sensitivity cardiac troponin T.

Only 12.5% of all patients required short-term sublingual nitrate for relief of angina during AAES, and of these, only 1 (25%) had reached the CPET angina threshold during the session. No significant differences occurred between hs-cTnT levels and nitrate use during AAES (p = 0.077) or between hs-cTnT levels and CCS functional class of angina of patients (p = 0.395). No adverse events occurred during AAES throughout the study.

## Discussion

To the best of our knowledge, this is the first study to specifically investigate MI by hs-cTnT after a single exercise session in patients with RA. Our results demonstrate that an AAES performed up to the angina threshold corresponding to 3 on the visual pain scale from 0 to 10 did not alter hs-cTnT concentration or cause clinical complications in patients with RA. Therefore, physical exercise may be more comprehensively indicated as part of nonpharmacological treatment of these patients.

These results are an important step in clinical practice, because one of the most important clinical characteristics of patients with RA is low exercise tolerance, limiting their daily activities and worsening their quality of life.^12^ In this context, physical exercise could be included as part of safe clinical treatment of RA. Habitually, these patients are restricted in physical activities because of the fear that exercise may exacerbate symptoms or trigger recurrent cardiovascular events. Cardiac rehabilitation, an established treatment for CAD patients^13,14^ (class I, level of evidence A), is not clearly recommended for patients with RA, because of the lack of evidence regarding the safety and beneficial effects.^15,16^

Hs-cTnT is a recognized biomarker for diagnosis of myocardial infarction and MI.^17,18^ Troponin concentrations, even below the 99th percentile, predict adverse outcomes in patients and the general population.^19^ In patients with CAD, including those with RA,^1^ hs-cTnT is an important prognostic marker of cardiovascular events.^1,20,21^ Low circulating hs-cTnT was identified as the strongest predictor of death and nonfatal MI. A possible explanation is due to the occurrence of silent ruptures of vulnerable atherosclerotic plaques, which lead to microembolization and subsequent microinfarction in areas of myocardium not supplied by sufficient collateral circulation, elevating hs-cTnT plasma levels before MI and cardiovascular death occurrence.^7^

Cardiac troponins can also be released with exercise; however, the underlying mechanisms and the real clinical value of this increase are not completely understood.^8,10,22^ Several theories have been proposed to explain the mechanism underlying troponin release, followed in many cases by documented echocardiographic ejection fraction abnormalities.^23^ MI is the most concerning proposed mechanism, because it could result in a negative clinical outcome. Another well accepted mechanism is the release of an unbound troponin that exists in cardiomyocyte cytosol through the increased membrane permeability of cardiomyocytes (triggered by the shear stress of exercise). This could explain the short duration (< 1 week) of hs-cTnT increase after exercise.

The magnitude of the hs-cTnT response depends on the duration and, principally, intensity of physical activity, and the long-term prognostic significance of repetitive, exercise-induced troponin release is not completely known.^10,22^ Many authors consider these exercise-induced increases in troponin concentrations benign, because they occur frequently, are present even in (apparently) healthy individuals, and are not accompanied by clinical symptoms.^24–26^ On the other hand, a recent study observed that baseline and postexercise hs-cTnT were independent predictors of mortality and major adverse cardiovascular events in a cohort of older long-distance walkers, calling attention to the fact that high troponin levels may not be only a benign physiological response, but an early prognostic marker of cardiovascular events.^19^

Considering the lack of consensus about the clinical relevance of postexercise hs-cTnT on prognosis, the interpretation of these studies must take the following into account: 1) population evaluated, 2) intensity and volume of exercise applied, 3) hs-cTnT measurement assays, 4) training experience, 5) blood sample timing, and 6) presence of symptoms during exercise.^10,22^

In view of these controversial points and our findings, it seems that physical activity, especially in patients with CAD, may be safely practiced when it is not followed by an increase in postexercise hs-cTnT, guiding the prescription of an “optimal target dose.” Accordingly, it minimizes the possible harmful effects of exercising, achieving maximal health benefits, and additionally changing paradigms of limiting exercise prescription below ischemia or angina threshold.

Physical training based on the pain threshold associated with the visual pain scale is widely used and accepted in patients with peripheral arterial disease.^27^ In these patients, this type of training is considered a form of treatment.^27^ In patients with CAD, exercise is actually recommended at the intensity below inducible ischemia. The aerobic exercise intensity should be prescribed 10 beats below the ischemia threshold, usually controlled by electrocardiographic changes.^7^ In patients with RA, who are extremely limited in performing daily tasks due to angina and whose symptoms are triggered by both physical and emotional stressors, it is a challenge to define exercise-based HR.

In our patients with RA, 25% had hs-cTnT above the 99th percentile of normality (> 14 ng/L), that is, at a higher risk of cardiovascular events. In spite of this and the presence of moderate angina (up to 3 of 10 on visual pain scale), in more than a half of the patients, we did not observe a significant increase in hs-cTnT after AAES or clinical events during the session. Angina, like other chronic pain, is a subjective and very individual symptom and it should, of course, be taken into consideration for the safety of these patients. It is essential to highlight that our patients did not experience electrocardiographic alterations during the exercise session, even though they experienced angina. Therefore, an exercise intensity prescription based on electrocardiographic modifications does not appear to be an appropriate strategy in patients with RA.

We have to emphasize that patients who experienced angina during the exercise session had higher HR than patients without angina. However, the patients without angina trained to the anaerobic threshold or moderate effort, which is the recommended exercise intensity for CAD patients. These results reinforce that our proposed exercise prescription may be considered safe to apply in patients with RA.

### Limitations

The hs-cTnT analysis was made based on 2-point samples: before and 3 hours after an AAES. The last one was defined based on evidence that any change in hs-cTnT level is already detectable at that time;^17,28^ therefore, we do not believe that it interfered with our results.

## Conclusion

A single AAES performed at the angina threshold and corresponding visual pain scale (up to 3 of 10) did not alter hs-cTnT in patients with RA, suggesting that no significant MI was elicited by exercising. Therefore, we conclude that this exercise protocol can be considered safe for patients with RA.

### What is known about the topic and what does this study add?

Exercise training improves exercise tolerance and quality of life and reduces mortality in this group of patients; however, the safety of physical activity is still questioned for patients with refractory angina and is often an impediment to performing cardiac rehabilitation, thus impacting this population's quality of life. This manuscript creates a paradigm for future studies of rehabilitation in patients with refractory angina.
